# Single nuclei transcriptomics of the in situ human limbal stem cell niche

**DOI:** 10.1038/s41598-024-57242-4

**Published:** 2024-03-21

**Authors:** Kathryn C. Davidson, Minkyung Sung, Karl D. Brown, Julian Contet, Serena Belluschi, Regan Hamel, Aida Moreno-Moral, Rodrigo L. dos Santos, Julian Gough, Jose M. Polo, Mark Daniell, Geraint J. Parfitt

**Affiliations:** 1 Department of Anatomy and Developmental Biology, Monash University, Clayton, VIC Australia; 2 Mogrify Limited, Cambridge, England, UK; 3 Centre for Eye Research Australia (CERA), Melbourne, Australia; 4 MRC Laboratory of Molecular Biology, Cambridge, England, UK; 5 Development and Stem Cells Program, Monash Biomedicine Discovery Institute, Clayton, VIC Australia; 6 Australian Regenerative Medicine Institute, Monash University, Clayton, VIC Australia; 7 Adelaide Centre for Epigenetics, Faculty of Medicine Nursing and Medical Sciences, The University of Adelaide, Adelaide, Australia; 8 The South Australian Immunogenomics Cancer Institute, Faculty of Medicine Nursing and Medical Sciences, The University of Adelaide, Adelaide, Australia; 9 Ophthalmology Discovery Research, AbbVie, Irvine, CA USA

**Keywords:** Adult stem cells, Quiescence, Stem-cell differentiation, Stem-cell niche

## Abstract

The corneal epithelium acts as a barrier to pathogens entering the eye; corneal epithelial cells are continuously renewed by uni-potent, quiescent limbal stem cells (LSCs) located at the limbus, where the cornea transitions to conjunctiva. There has yet to be a consensus on LSC markers and their transcriptome profile is not fully understood, which may be due to using cadaveric tissue without an intact stem cell niche for transcriptomics. In this study, we addressed this problem by using single nuclei RNA sequencing (snRNAseq) on healthy human limbal tissue that was immediately snap-frozen after excision from patients undergoing cataract surgery. We identified the quiescent LSCs as a sub-population of corneal epithelial cells with a low level of total transcript counts. Moreover, TP63, KRT15, CXCL14, and ITGβ4 were found to be highly expressed in LSCs and transiently amplifying cells (TACs), which constitute the corneal epithelial progenitor populations at the limbus. The surface markers SLC6A6 and ITGβ4 could be used to enrich human corneal epithelial cell progenitors, which were also found to specifically express the putative limbal progenitor cell markers MMP10 and AC093496.1.

## Introduction

The cornea is a transparent connective tissue at the front of the eye that focuses light onto the lens and retina as part of the visual pathway. The corneal epithelium is the most anterior layer of the cornea which retains the tear film on the ocular surface and prevents pathogens entering the eye. Corneal epithelial cells are continuously renewed in a centripetal fashion, from periphery to central cornea, by quiescent limbal stem cells (LSCs)^1–6^. Injury to the limbus can result in Limbal Stem Cell Deficiency (LSCD), an orphan disease typically caused by ocular burns, although it can also be a consequence of diseases such as aniridia or Stevens–Johnson syndrome. When LSCD occurs from trauma, the conjunctiva overgrows the cornea surface due to the depletion of the LSC population and limbal barrier, ultimately leading to corneal opacity and blindness^7^.

It was first shown that LSCs are a quiescent adult stem cell population through label-retention studies that used thymidine radio-labelling^1^. Lineage tracing of corneal epithelial cells, driven by the keratin 14 promoter in transgenic mice, confirmed the centripetal renewal of central corneal epithelial cells by LSCs at the peripheral limbus^2,3^. Importantly, LSC transplantation for LSCD was the first autologous stem cell therapy approved for use in Europe, as Holoclar® was granted market authorization by the EMEA in 2015^8^. However, the identification of markers for LSC purification remains of high clinical value as pure LSCs could be used to generate off-the-shelf corneal epithelial cell sheets for LSCD therapy with a greater capacity for long-term regeneration of the corneal epithelium. There is also a need to better understand the transcriptomic changes associated with adult stem cell quiescence and how they regulate asymmetric division.

Previous research has focused on elucidating LSC markers for their purification and clinical application^5,9–13^; however, LSC markers remain elusive. Some of the markers that have classically been used to identify LSCs include ATP transporters such as ABCB5 and ABCG2. For example, ABCB5 has been suggested as an important LSC marker and is being used in clinical studies for the purification of LSCs for the treatment of LSCD^14^. Putative LSC markers also include ENO-1, TP63 (DeltaNp63a isoform), BMI1, and FZD7^15–18^ and more recently, MMP1, MMP10, GPHA2, AC093496.1, and CASP14 have been suggested as progenitor markers from single cell RNA sequencing studies using corneal epithelial cells derived from cadaveric limbus tissue^19,20^. Adult stem cell quiescence has also been exploited as a functional marker of LSCs through pulse-chase labelling of GFP label-retaining cells with doxycycline in H2B-GFP/K5tTA transgenic mice^4,6^.

In this study, we carried out single nuclei RNA-sequencing (snRNAseq) of snap-frozen human limbus tissue from cataract patients to unbiasedly reveal the cellular heterogeneity in the corneal-limbus epithelium and determine the transcriptional profile of the different corneal epithelial cells, including LSCs. This allowed us to characterize the human corneal epithelium sub-populations and define the gene expression profiles of the different ocular surface epithelial cell types with the in situ transcriptome profile preserved. Finally, we confirmed the clonogenic potential of purified ITGβ4 + and SLC6A6 + cells in comparison to unpurified cells using colony and holoclone forming efficiency assays.

## Results

### Single nuclei RNA-sequencing of healthy limbus tissue snap frozen from cataract patients

**Figure 1:**
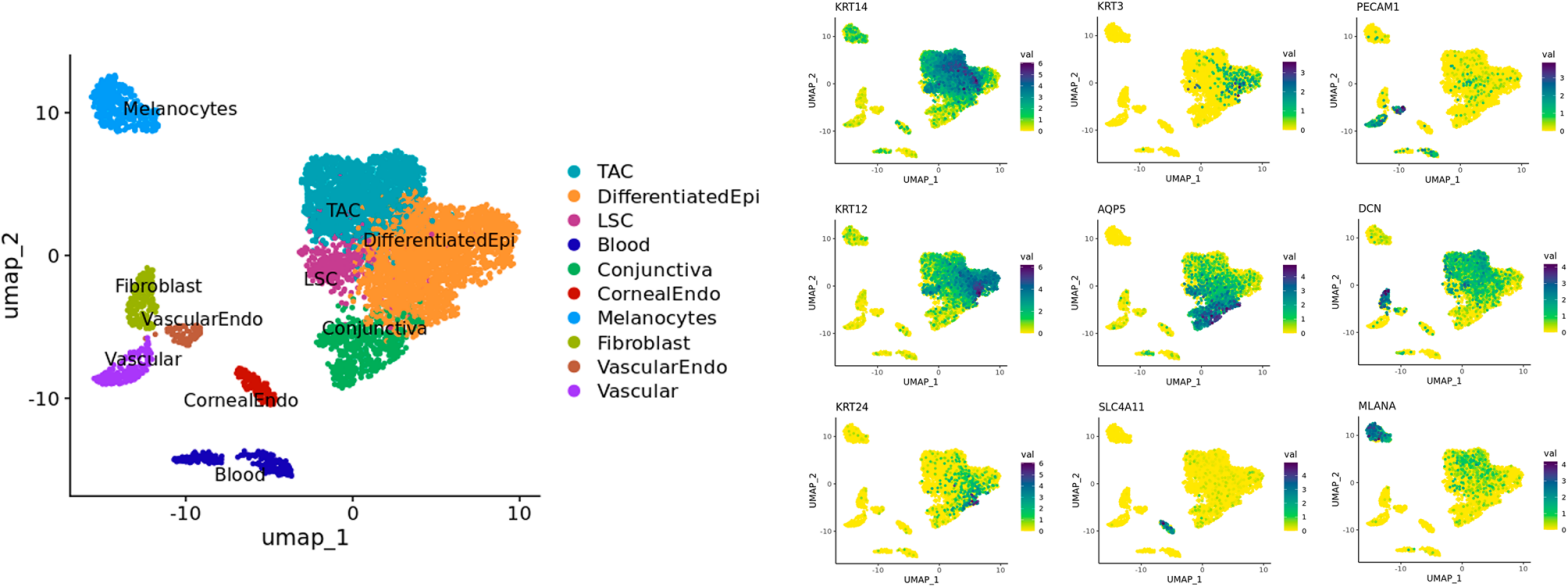
Cell clustering and annotation of single nuclei RNAsequencing data of human limbus samples snap-frozen from cataract patients (n = 10). Separate clusters were identified for basal corneal epithelial cells (KRT14), differentiated corneal epithelial cells (KRT12 and KRT3), supra-basal corneal epithelial cells (KRT24), conjunctival epithelial cells (AQP5), corneal endothelial cells (SLC4A11), vascular endothelial cells (PECAM1), stromal fibroblasts (DCN), and melanocytes (MLANA) based on the expression of well-established cell specific markers.

We used snRNAseq to obtain an unbiased transcriptional profile of the different cells of the corneal epithelium, as snRNAseq has previously been used to resolve the cellular heterogeneity and transcriptional profiles of several frozen tissues and organs. To preserve the in situ transcriptomic profile of the limbus, healthy human limbal tissue was excised from cataract patients for immediate snap-freezing with liquid nitrogen. SnRNAseq was then performed using the 10X genomics chromium platform on 10 pooled human limbus samples to define the transcriptomes of the cell sub-populations within the human limbus (Fig. 1).

The Uniform Manifold Approximation and Projection (UMAP) algorithm was used for dimension reduction to visualize how separable the cell clusters are with respect to their transcriptomes. For cell annotation of the limbus sub-populations in the UMAP plot (Fig. 1), cytokeratin (KRT) expression was used to define the basal (KRT14) and differentiated populations of the cornea (KRT12 and KRT3). Supra-basal corneal epithelial cells (KRT24), conjunctival epithelial cells (AQP5), corneal endothelial cells (SLC4A11), vascular endothelial cells (PECAM1), stromal fibroblasts (DCN), and melanocytes (MLANA) were also used to annotate cell type based on well-established markers.

**Figure 2:**
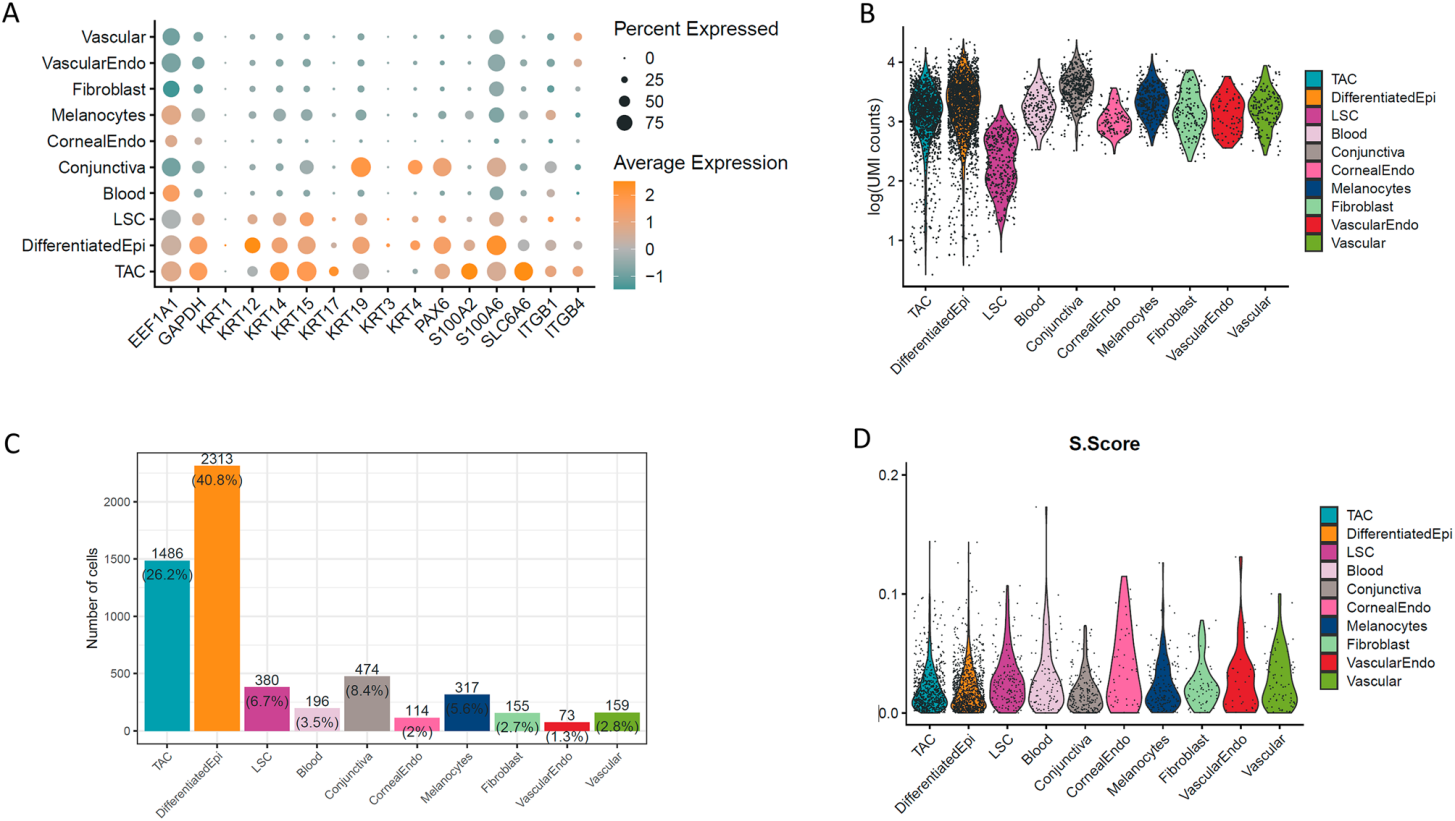
Expression profiles, cell frequencies, total read counts, and cell cycle S scores across ocular surface cell sub-populations. (**A**) In terms of cytokeratin expression, LSCs and TACs progenitors highly express KRT14 and KRT15, while TACs are enriched for KRT17 and differentiated corneal epithelial cells exclusively express KRT3 and KRT12. Conjunctival epithelial cells express KRT4. S100A2 was expressed in TACs, whereas S100A4 and S100A6 were enriched in differentiated corneal epithelial cells and conjunctival epithelial cells. ITGβ1 and ITGβ4 are highly expressed in approximately 25% of LSCs and 50% of the TAC population. (**B**) Total read counts represent the level of gene expression in each cell type captured, which was used to identify LSCs as a basal corneal epithelial cell population with a low-level of gene expression. (**C**) Cell frequencies indicate that approximately 6.7% of sequenced cells were identified as LSCs. (**D**) The S score was used to assess cell cycle rate in the cell clusters by the expression level of markers of the DNA synthesis (S-phase) stage of cell division, however, no discernable sub-population of quiescent LSCs with a low S score could be readily identified.

The LSC cluster was defined as the remaining cluster with a gene expression profile closest to basal corneal epithelial cells, and one differentiator was the global suppression of their transcriptome (Fig. 2), which is likely tied to their quiescent state. To specifically identify the LSC cluster in the UMAP plot, we assessed the expression of epithelial cytokeratins (KRT3; KRT12; KRT14; KRT15); and S100 genes (Fig. 2A); which have previously been used as markers to identify LSCs^6,15,21–23^. We also determined total gene expression levels (Fig. 2B), cell type frequency (Fig. 2C), and the S score, which is used to determine the level of cell division (Fig. 2D). KRT15, ITGβ1, and ITGβ4 represented some of the highest transcript levels in the LSC sub-population, and these genes were found expressed in approximately 50% (KRT15), and 25% (ITGβ1 and ITGβ4) of cells within the LSC cluster (Fig. 2A). KRT17 increased in expression as LSCs become transiently amplifying cells (TACs) that frequently divide, while KRT12, S100A4 and S100A6 increase in expression as TACs proliferate and differentiate to corneal epithelial cells. The LSC population represented 6.7% of the total cell population that was sequenced (Fig. 2C), however, there was no definitive quiescent sub-population of the LSCs from the S score (Fig. 2D).

**Figure 3:**
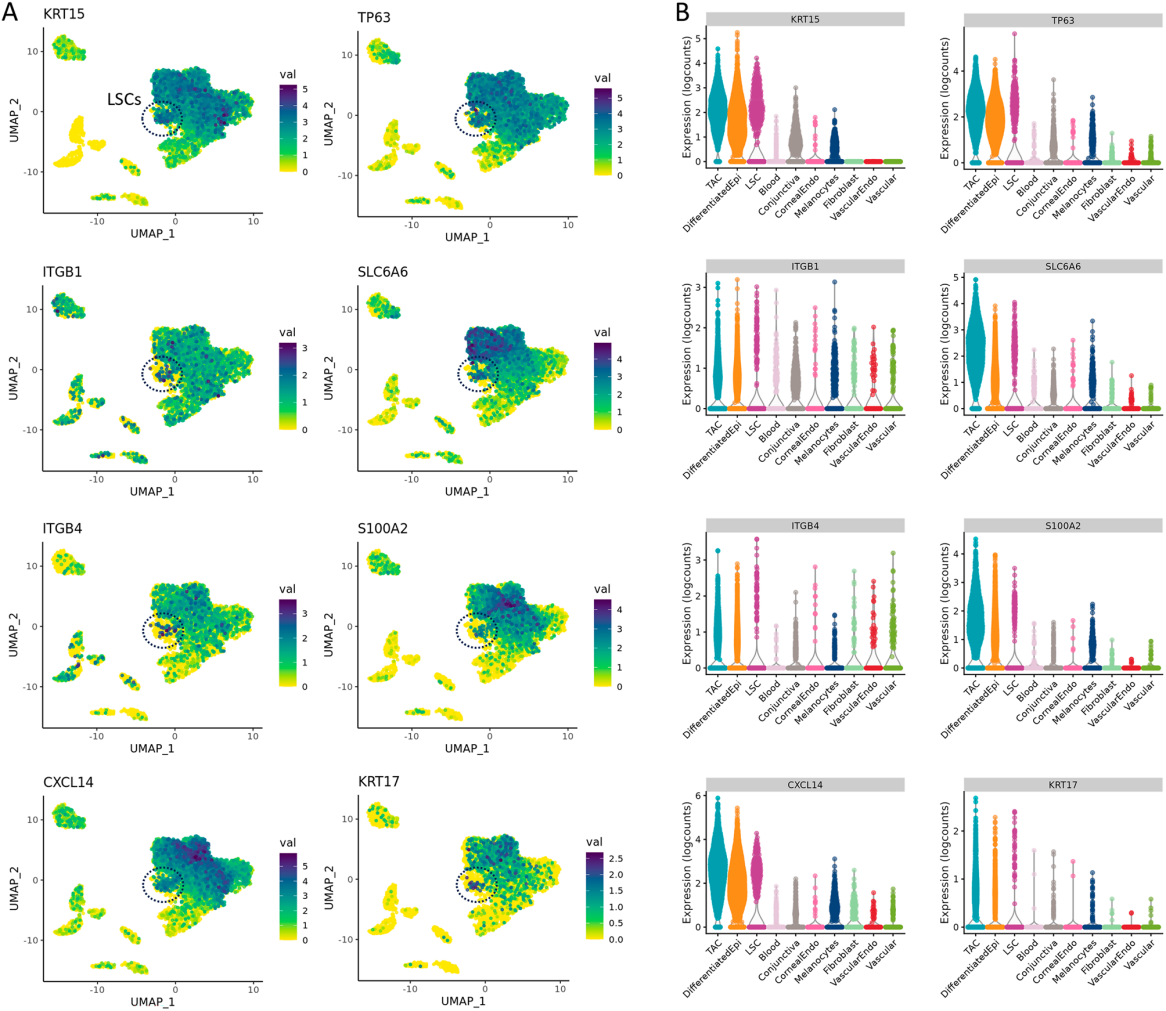
Expression profiles of genes highly expressed in LSC and TAC progenitors of the corneal epithelium. (**A**) Localization and (**B**) expression levels of KRT15, TP63, ITGβ1, SLC6A6, ITGβ4, S100A2, CXCL14, and KRT17 in corneal epithelial cell types. KRT15, TP63, CXCL14, and ITGβ4 were found to be highly expressed in both LSCs and TACs. S100A2 and SLC6A6 were largely expressed in the TAC progenitor population.

We investigated the genes specific to the LSC and TAC sub-populations and found KRT15, CXCL14, ITβ4, and TP63 were highly expressed in these progenitor populations of the limbus (Fig. 3). SLC6A6 expression was found to be more specific to the LSCs and the TAC population, which may represent a viable candidate to enrich corneal epithelial progenitor populations as a surface marker, alongside integrin β4. S100A2 was also found to be expressed higher in TACs when compared with differentiated corneal epithelial cells. Interestingly, CXCL14, S100A2, and SLC6A6 expression was largely absent from the conjunctival epithelium.

**Figure 4:**
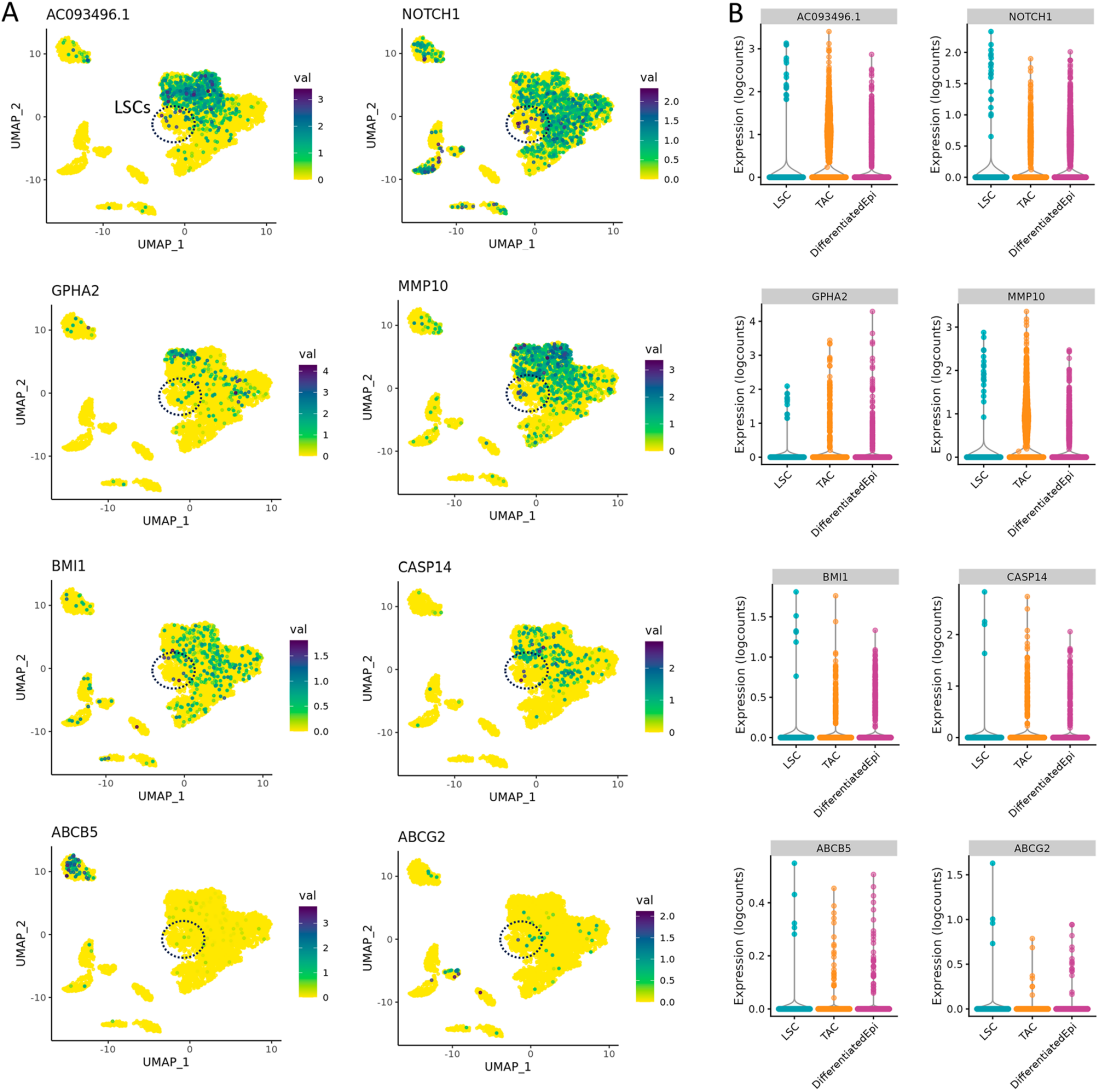
Expression profiles of putative LSC markers in the limbus. (**A**) Localization and (**B**) expression levels of AC093496.1, NOTCH1, GPHA2, MMP10, BMI1, CASP14, ABCB5, and ABCG2. We found that AC093496.1 and MMP10 expression was largely specific to the TACs sub-population and may represent the best markers for corneal epithelial cell progenitors. GPHA2 was expressed in a sub-set of TACs and differentiated corneal epithelial cells. A small proportion of the LSC cluster showed NOTCH1 expression, which was widely expressed in other epithelial cell types, much and like BMI1 and CASP14. We did not observe high expression of either of the ATP transporters, ABCB5 or ABCG2, in the LSC or TAC progenitor populations. In fact, ABCB5 expression was confined to the melanocyte population in our dataset.

We also determined the expression levels of putative LSCs markers, such as AC093496.1, NOTCH1, GPHA2, MMP10, CASP14, and ABCB5 (Fig. 4). In our data, AC093496.1 and MMP10 are specifically expressed in the progenitor TAC population and could be used as markers, alongside SLC6A6. GPHA2 was found to be present in a sub-population of TACs and differentiated corneal epithelial cells, while NOTCH1 had the highest expression level in LSCs and low-level expression throughout the corneal and conjunctival epithelium. BMI1 and CASP14 did not show any meaningful expression levels in the LSC and TAC populations, and ABCB5, which has been suggested as an LSC marker, was found to expressed only in melanocytes in our dataset.

### Immunolabelling and holoclone forming efficiency of purified SLC6A6 and ITGβ4 positive cells from cadaveric human limbus tissue

**Figure 5:**
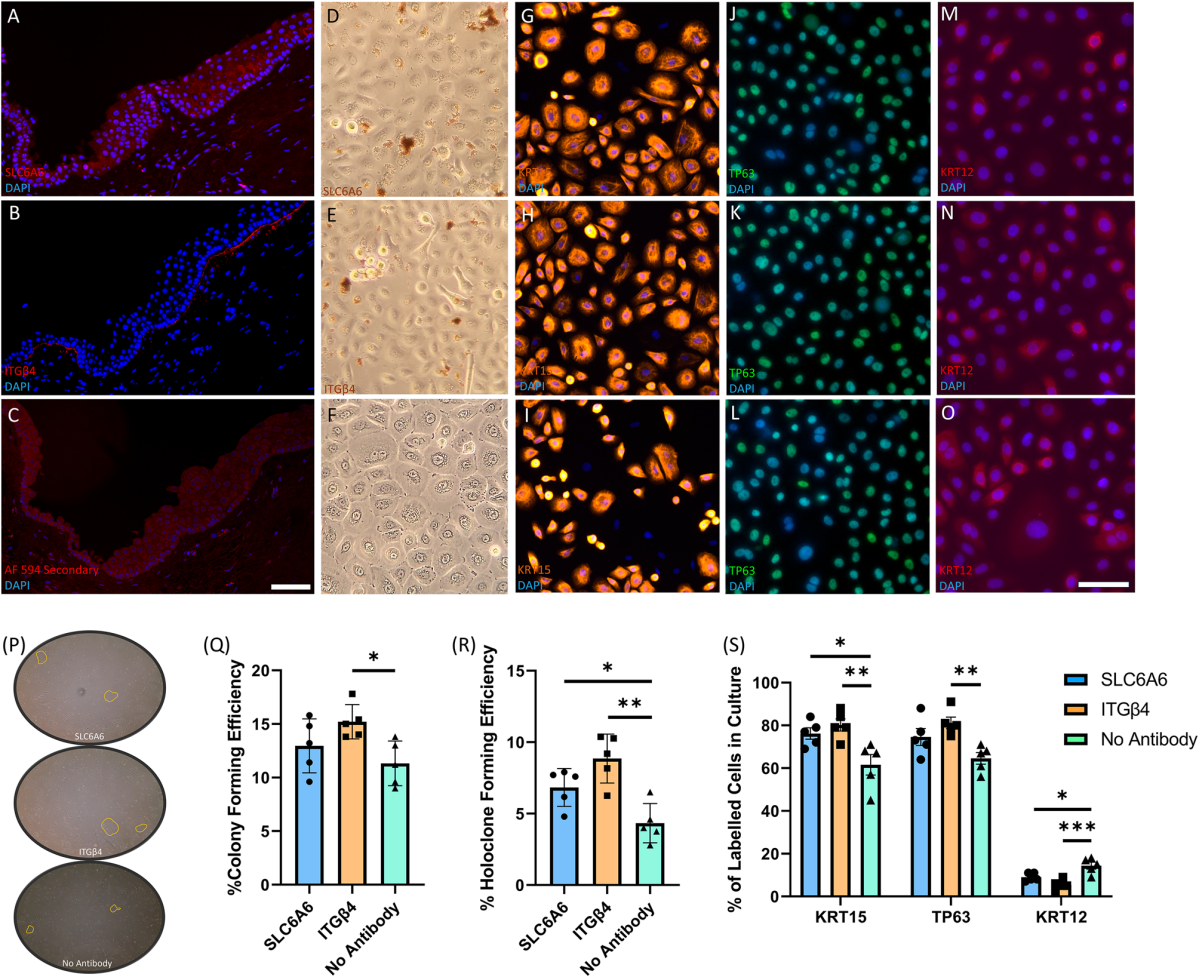
Immunofluorescence and in vitro validation of SLC6A6 and ITGβ4 surface markers of limbal epithelial progenitor cells in cadaveric human tissue. Cadaver human limbus tissue sections were stained with (**A**) SLC6A6, (**B**) ITGβ4, or (**C**) secondary antibody alone, to identify if these putative progenitor surface markers were expressed at the limbus, as suggested by the snRNAseq data. SLC6A6 was found to be expressed in basal and suprabasal limbal epithelial cells, while ITGβ4 was limited to basal expression at the limbus, and no specific antibody labelling was observed when only the secondary antibody was used. (**D**) SLC6A6 antibodies conjugated to magnetic beads were able to isolate limbal progenitor cells that can be expanded in CnT-Prime media to reach confluence in 24-well plates within 8 days. Immunolabelling of SLC6A6 purified cultures for KRT15 (**G**), TP63 (**J**), and (**M**) KRT12 confirmed their progenitor capacity in vitro. (**E**) ITGβ4 conjugated antibodies are also able to isolate limbal progenitors that expand and reach confluence in vitro and can be immunolabelled for for KRT15 (**H**), TP63 (**K**), and (**N**) KRT12. (**F**) Limbal epithelial cells that were surgically extracted from cadaveric limbal biopsies and not enriched by surface markers also reach confluence within 8 days, however, they exhibit a different immunofluorescence profile compared to SLC6A6 and ITGβ4 purified cells when stained for KRT15 (**I**), TP63 (**L**), and (**O**) KRT12 (Scale bar = 100µm). (**P**) Colonies present in culture after 10 days from seeding were quantified using ImageJ software and used to determine the colony formation efficiencies in (**Q**). (**R**) Colonies were isolated, dissociated, and then re-plated into individual wells to determine holoclone forming efficiency, which was calculated by multiplying the original colony formation efficiency with the percentage of re-seeded wells with a holoclone present. (**S**) The number of cells that express KRT15, TP63, and KRT12, was quantified in SLC6A6, ITGβ4, and unpurified cultures by immunocytochemistry after they had reached confluence.

To validate the snRNAseq of human limbus biopsies from cataract patients, we performed immunohistochemistry, immunocytochemistry, and cell purification for in vitro clonogenicity assays using the limbal progenitor markers SLC6A6 and ITGβ4 identified through snRNAseq (Fig. 5). In Fig. 5A, SLC6A6 is shown to be expressed in the membranes of basal and suprabasal limbal epithelial cells in cadaveric human tissue sections, whereas ITGβ4 was found to be expressed on the basal side of the human limbus (Fig. 5B). When only secondary antibody was used on human limbus sections, no staining was apparent (Fig. 5C).

Importantly, we were able to isolate SLC6A6 + (Fig. 5D,G,J,M), and ITGβ4 + (Fig. 5E,H,K,N) cells using antibodies directly conjugated to magnetic beads and then purified using an ‘easysep’ magnet for 10min for in vitro assay. Unpurified cells dissociated straight from cadaveric tissue are shown in Fig. 5F,I,L,O. Next, we compared the proliferation potential of purified and unpurified cells over 10 days and found that they all maintained their capacity to reach confluence when 25,000 cells were seeded into 24-well plates with CnT-Prime media. Immunocytochemistry was then used to compare the expression of limbal (KRT15—Fig. 5G–I), basal (TP63—Fig. 5J–L), and differentiated corneal epithelial cell markers (KRT12—Fig. 5M–O) in SLC6A6 + and ITGβ4 + purified cells compared with unpurified cells.

To determine the colony and holoclone forming efficiency of unpurified cells and SLC6A6 + and ITGβ4 + purified cells from cadaveric limbal biopsies, we plated 500 cells into 6-well plates and cultured in CnT-Prime for 8 days to quantify the number of colonies present (Fig. 5P). We found that colony forming efficiency (CFE) (Fig. 5Q) was significantly greater in ITGβ4 + purified cultures in comparison to unpurified cells (ITGβ4 + CFE = 15.2% ± 0.71 versus unpurified CFE = 11.32% ± 0.93, *P* = 0.031), as well as holoclone forming efficiency (Fig. 5R—ITGβ4 + HFE = 8.9% ± 0.76 versus unpurified HFE = 4.33% ± 0.0.62, *P* = 0.001). However, there was no significant difference in colony forming efficiency between SLC6A6 purified cells and unpurified cells, although there was a significant difference in holoclone forming efficiency when SLC6A6 cultured cells were compared with unpurified cells (SLC6A6 HFE = 6.83% ± 0.59 versus unpurified HFE = 4.33% ± 0.0.62, *P* = 0.049). Taken together, these results indicate that ITGβ4 + is capable of labelling progenitor cells with a higher colony and holoclone forming potential than unpurified cells.

After magnetic isolation of SLC6A6 and ITGβ4 cells, we also cultured 25,000 cells in 24-well plates and CnT-Prime media for 8 days before fixation in paraformaldehyde and immunocytochemistry labelling for KRT15, TP63, and KRT12, to determine the extent of basal and differentiated corneal epithelial cells in culture (Fig. 5S). Of note, we found that ITGβ4 + purified cells generate a significantly greater proportion of the basal limbal epithelial markers KRT15 (ITGβ4 + KRT15 = 80.2% ± 2.85 versus unpurified KRT15 = 61.6% ± 4.77, *P* = 0.0079) and TP63 (ITGβ4 + TP63 = 81.2% ± 5.9 versus unpurified TP63 = 64.6% ± 6.025, *P* = 0.0077). Moreover, the percentage of cells that express the differentiated corneal epithelial marker KRT12 was significantly decreased in ITGβ4 + purified cells (ITGβ4 + KRT12 = 5.4% ± 1.12 versus unpurified KRT12 = 14.4% ± 1.6, *P* = 0.0006), suggesting that ITGβ4-positive cells preferentially maintain an undifferentiated state when proliferating.

### Limbal stem cell differentiation to corneal epithelium unveiled by trajectory analysis of gene expression

**Figure 6:**
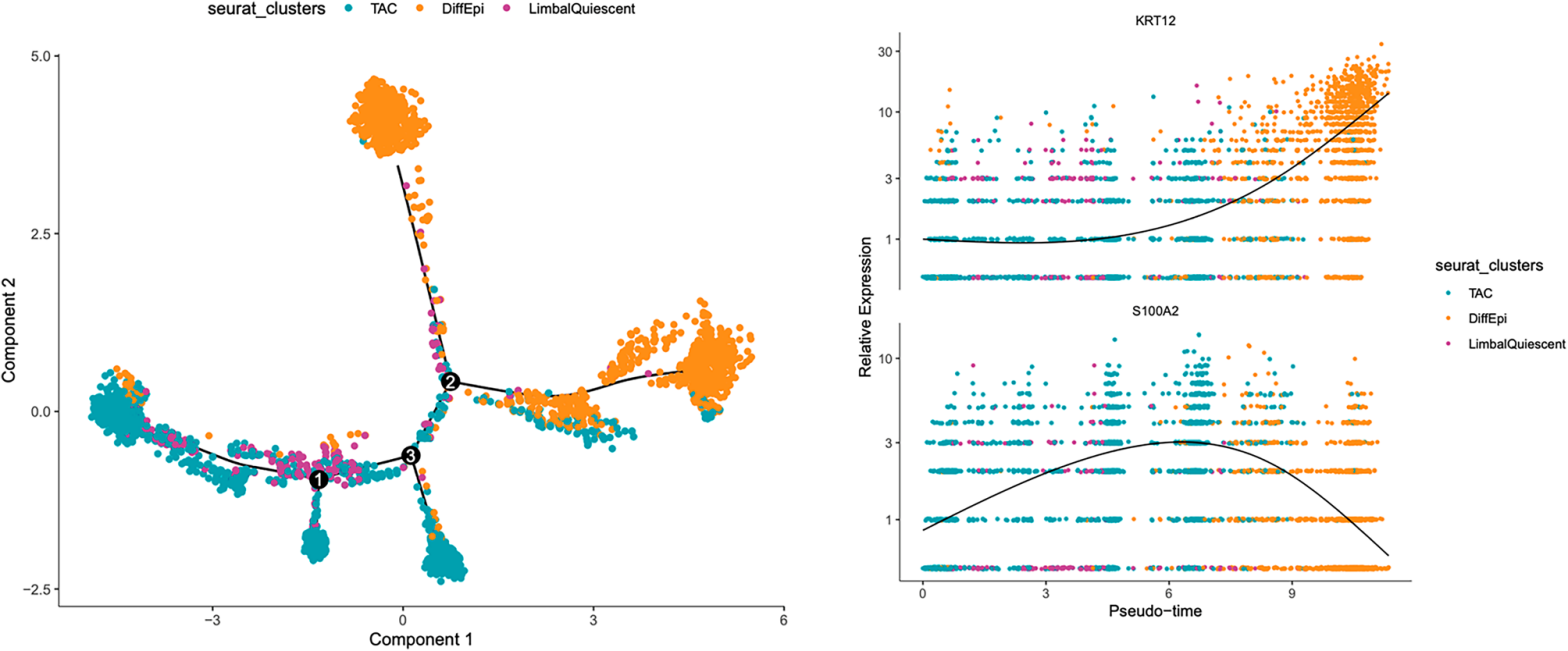
Pseudo-time analysis of LSC differentiation to corneal epithelial cells. LSCs (purple) and TACs (cyan) exhibit a similar gene expression pattern and overlap in a trajectory analysis of LSC differentiation, suggesting that LSCs are uni-potent progenitors of TACs. Differentiated corneal epithelial cells (orange) are defined by increased KRT12 expression and a loss of S100A2. Overall, LSC differentiation is defined by a loss of stem cell quiescence, centripetal migration towards the central cornea and a subsequent increase in KRT12 expression and loss of S100A2 expression.

To determine the gene expression changes that define LSC differentiation, we applied a pseudo-time analysis to understand the trajectory and gene expression changes that occur as LSCs transition to differentiated corneal epithelial cells. Pseudo-time analysis is a computational method that infers a dynamic trajectory of a process, such as cell differentiation, from a snapshot of cells in different states of the entire process. In this way, the pseudo-time analysis can capture a biologically relevant process in the dataset, such as a progression from stem cells to terminally differentiated epithelial cells in our case (Fig. 6). In this way, we aimed to determine the genes that may be responsible for stem cell quiescence or activation of LSCs to undergo differentiation to corneal epithelial cells.

In Fig. 6, the cells that we have identified as LSCs in our samples are inferred to be at one end of the pseudo-time projection, and as this is an unsupervised analysis, these results support our initial conclusion with respect to that cell population. Secondly, we found that the most distinctive gene expression changes that occurred when LSCs differentiated to corneal epithelial cells was an increase in KRT12 coupled with a decrease in S100A2. However, S100A2 was expressed in LSCs and TACs so the two populations have merged when it is used to project cell differentiation. The similarity in the transcriptomes of LSCs and TACs was observed in the trajectory analysis as the clusters consistently overlap. From this, we can infer that LSCs and TACs have similar transcriptomes and, therefore, LSC quiescence may be regulated through epigenetic changes, paracrine cell signaling, the extra-cellular matrix that forms the stem cell niche, or in development.

## Discussion

From the snRNAseq data of snap frozen human limbus tissue, we have determined that the transcriptome of LSCs is largely suppressed when compared to other corneal epithelial cells and the transcriptome profile remains largely unchanged as LSCs give rise to their progeny. In 10 pooled human limbus samples, 380 (6.7%) nuclei were identified as LSCs based on their low level of gene expression and similarity to basal corneal epithelia (TAC) gene expression. LSCs and TACs were also found to have a similar gene expression profile to bulbar conjunctival epithelial cells adjacent to the limbus, however we were able to discern sub-population of cells with established markers, such as the cytokeratins 3, 12, 14, 15, and 24. The suppression of LSC gene expression may be explained by the fact that a large proportion of the most quiescent LSCs may serve as a reservoir that is only triggered to proliferate in the event of injury, as corneal wounding studies have shown a seven-fold increase in the number of LSCs undergoing mitosis^24^.

A potential limitation of our study is the bias towards limbal epithelial cell types at the cornea periphery. Due to the excision of 2mm^2^ of limbus tissue, there is likely to be an underrepresentation of fully differentiated central corneal epithelial cells that are generated through centripetal renewal from the limbus. Another limitation is the donor age (mean = 68 years) may have impacted the viability, frequency, and transcriptome profile of limbal stem cells when compared to those isolated from young donors.

A major issue in the limbal stem cell field is the absence of a single LSC marker that enables their purification for further research and clinical application. ABCB5 has previously been suggested as an LSC marker^14^, however it was found to be specifically expressed in melanocytes in our dataset. Here, we show that the expression of key markers such as CXCL14, SLC6A6, ITGβ4, MMP10, AC093496.1, KRT15, and S100A2, best represents the TAC progenitor population when using 10X snRNAseq of the limbal stem cell niche frozen immediately after excision. Therefore, functional quiescence remains the most clear single marker of limbal stem cells in animal models, which cannot currently be translated to human tissue without in vitro expansion of the LSC population and the loss of quiescence.

**Figure 7:**
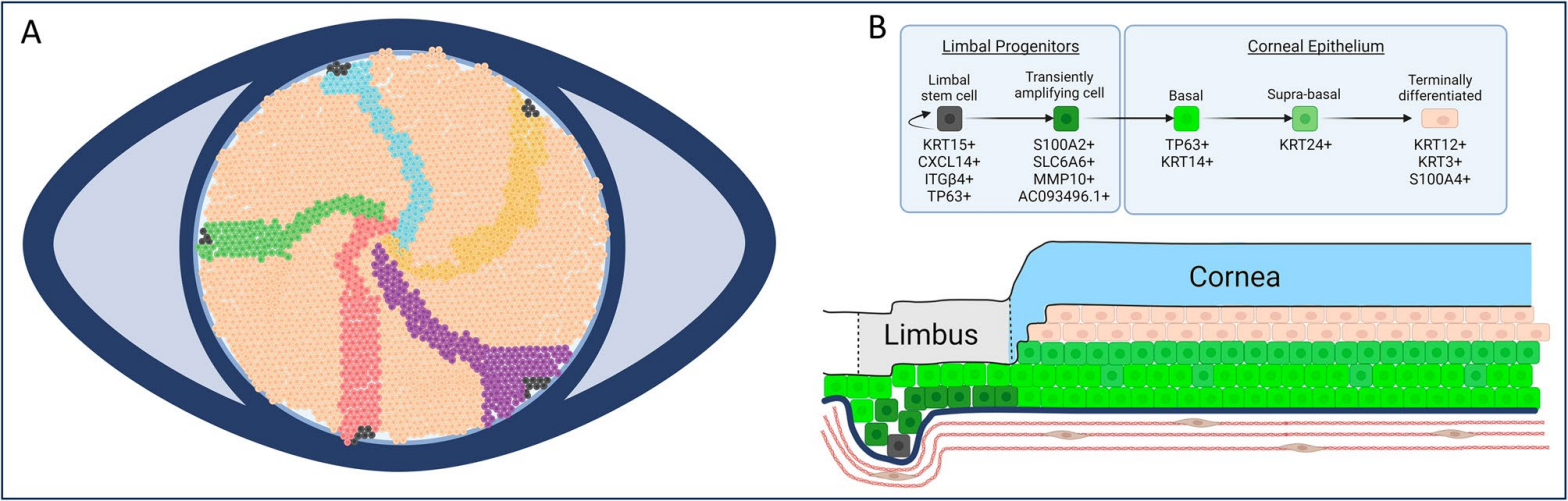
Schematic of LSC renewal of the corneal epithelium. (**A**) Quiescent LSCs (black) at the limbus and corneal periphery are uni-potent progenitors of the corneal epithelium that can self-renew to maintain a stem cell population. They give rise to the corneal epithelial cells in the centre in a centripetal fashion, as has been shown in lineage tracing and label-retention studies. (**B**) LSCs self-renew and give rise to transiently amplifying progenitor cells, which express the markers MMP10, AC093496.1, S100A2, and SLC6A6. As basal corneal epithelial cells become supra-basal cells, they express KRT24 before terminally differentiating and expressing KRT12 and KRT3.

Single cell transcriptomic analysis of human limbus tissue from cadaveric donor samples has already pointed to the importance of AC093496.1, MMP10, KRT15, ITGβ4, and S100A2 genes^25,26^ and, therefore, these genes may represent the most specific corneal epithelial progenitor cell marker panel. Keratins 15 and 17 have been shown to be highly expressed in quiescent limbal stem cells sequenced from transgenic mice^6^, whereas AC093496.1 is a long non-coding RNA with an unknown function. The gene expression changes that occur during LSC differentiation to corneal epithelium as defined by our snRNAseq data, are illustrated in Fig. 7. In short, we hypothesize that SLC6A6 and integrin β4 can likely be exploited as potential surface markers for the purification and expansion of corneal epithelial cell progenitors, which also co-express S100A2, MMP10, and AC093496.1.

MMP10 is a secreted matrix metalloproteinase involved in the break-down of extracellular matrix components, such as fibronectin, elastin, laminin, and various types of collagens. MMP10 overexpression slows the wound healing process in diabetic corneas^27^, which points to a role for MMP10 in regulating the renewal of the corneal epithelium. SLC6A6 is a membrane protein that transports taurine and β-alanine and is a member of a family of sodium- and chloride-ion dependent transporters. It has previously been identified as a limbal progenitor cell marker by single cell RNA sequencing of cadaveric tissue from young donors^28^.

Interestingly, CXCL14 was expressed in the limbus but was mostly absent from the conjunctiva. It is an epithelial chemokine that is broadly expressed and has shown to be important for embryogenesis and hematopoiesis^19^. CXCL14 has been shown to be expressed in the mouse limbus in development and adulthood, where it has been suggested that it may regulate the formation of limbal stem cells^22^. It has also been shown to regulate cell proliferation, invasion, and migrations of oral squamous carcinomas^29^, which may point to a role in the modulation of epithelial stem cell cycle rate. Additionally, it has been shown to have an important role in the inhibition of angiogenesis^30^, so it may help define the border between cornea and conjunctiva. Here, we find it expressed in the putative LSC population and the TACs of the basal corneal epithelium.

ITGβ1 has previously been suggested as marker of basal corneal epithelial cells and has been used to identify putative LSCs derived from iPSCs^15^. ITGβ1 − / − mice also have reduced corneal epithelial cell layers, alongside stromal defects^31^. ITGβ4 has previously been used to purify corneal epithelial cells derived from iPSCs that were expanded and used to treat patients with limbal stem cell deficiency, which was the first clinical use of iPSC-derived corneal epithelial cells^23^. Our study confirms that ITGβ4 may be used to help purify the basal population of the limbus and corneal epithelium to enrich corneal epithelial progenitors, as we found that purifying ITGβ4 positive cells from cadaveric limbal biopsies generates cells with a higher colony and holoclone formation when compared to unpurified cells. Moreover, purified ITGβ4 cells exhibit a significantly higher proportion of cells with TP63 and KRT15 immunolabelling than unpurified cells when allowed to reach confluence.

The S100 family of proteins are small (10-12kDa) calcium-binding cytosolic proteins that regulate calcium balance and have broad range of intra- and extracellular functions, including apoptosis and cell proliferation. S100A2 has previously been shown to be involved in limbal epithelial cell proliferation and differentiation and was found to be expressed in ocular surface squamous cell carcinomas. Importantly, in the same study S100A2 was shown to decrease with the expansion of LSCs in culture^21^. Using a pseudo-time analysis of gene expression changes across corneal cell types, we show here that a gradual decline in S100A2 expression correlates with an increase in KRT12 expression and corneal epithelial cell differentiation.

## Conclusion

In summary, we have been able to generate an unbiased transcriptional profile of the cornea limbus at single cell resolution and hypothesize that LSCs give rise to central corneal epithelial cells through an increase in KRT12 and KRT3 expression, alongside a decrease in TP63 and S100A2 expression. In agreement with recent transcriptomics studies of cadaveric limbus tissue, we also found that SLC6A6, MMP10, ITGβ4 and AC093496.1 localize to the transiently amplifying corneal epithelial progenitor population. We propose that SLC6A6 and ITGβ4 can be used as surface markers to enrich for corneal epithelial cell progenitors and that CXCL14 and S100A2 may play important roles in the regulation of LSC activation and quiescence; these genes can potentially be used as progenitor markers or clinical indicators of limbal stem cell deficiency. We confirmed the ability of ITGβ4 to mark corneal epithelial progenitors when compared to unpurified cells through colony and holoclone formation assays, as well as immunocytochemistry for basal limbal epithelial cell markers such as KRT15 and TP63, as well as the differentiated corneal epithelial marker, KRT12. However, the regulation of stem cell quiescence and gene suppression needs to be probed further to truly understand the control of adult stem cell activation and asymmetric division.

## Methods

### snRNAseq of human limbus tissue

To carry out snRNAseq of human limbus from patients (n = 10; 2 mm^2^ biopsy), tissue was extracted during cataract surgery and flash frozen on LN_2_ for preservation of gene expression profiles of the cell sub-types within the ocular surface epithelium. Samples were collected from 2 males and 8 females between the ages of 40 and 86 years of age (mean = 68 years; Table 1).

**Table 1:**
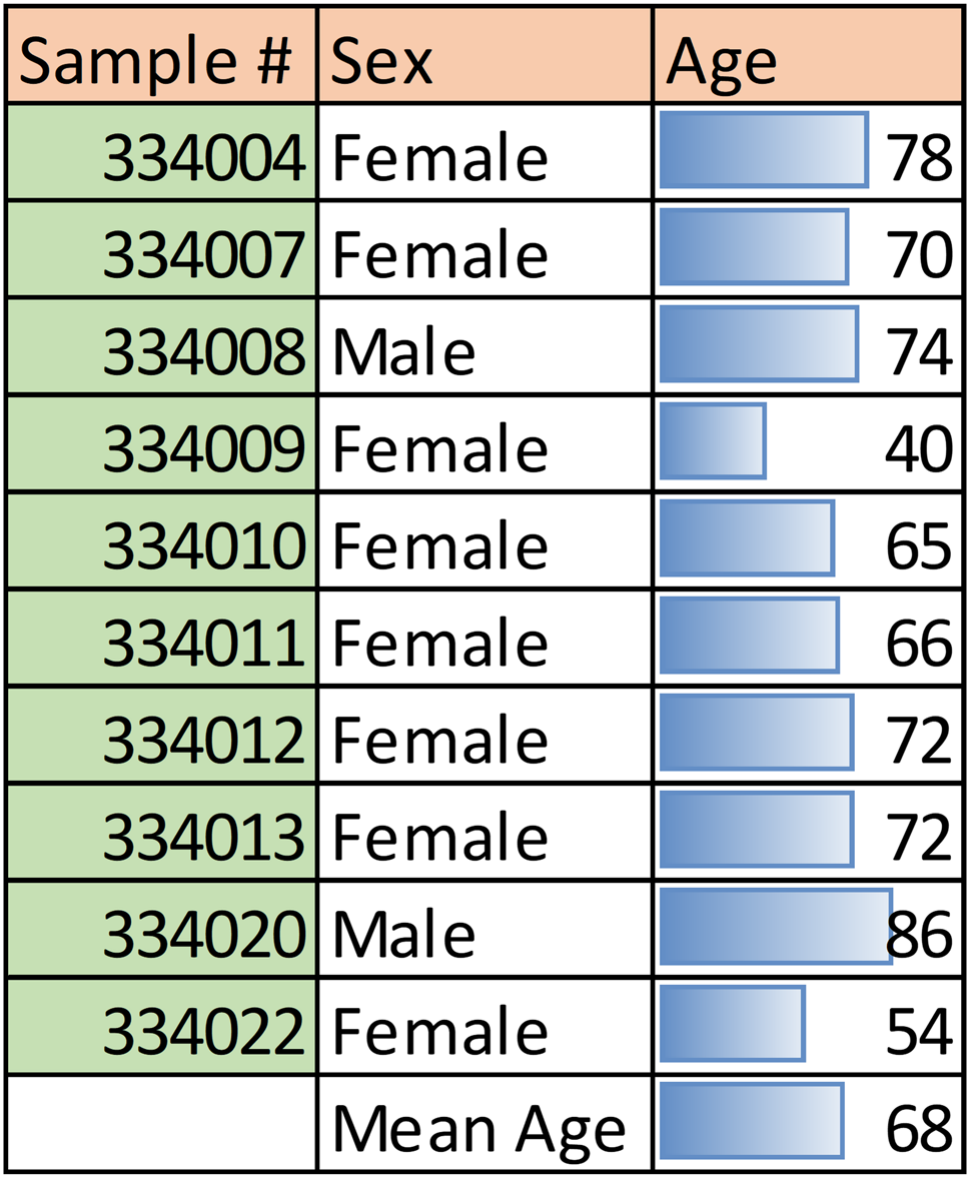
Age and sex of cataract patients who consented to excision and RNA sequencing of a 2 mm^2^ limbal biopsy.

Limbal samples were collected from 2 males and 8 females between the ages of 40 and 86 years of age (mean = 68 years).

Healthy human limbal tissue was collected from consenting cataract patients under the Royal Victorian Eye and Ear Hospital Human Research Ethics Committee approved study (ID:10/954H). Limbus tissue samples were snap frozen within 2 min of excision using LN_2_ were pooled and nuclei isolation was carried out using the Nuclei Isolation Kit: Nuclei EX Prep (Sigma, NUC101) as described previously (PMID 28,846,088). The short time from limbal excision to freezing prevents any possible gene expression changes that may occur before library preparation for snRNAseq.

All steps were carried out on ice. Briefly, tissue samples were homogenized using a glass Dounce grinder in 2 ml of ice-cold Nuclei EZ lysis buffer (Sigma N3408, with RNase inhibitor). Samples were transferred to a separate tube on ice while the glass grinder was rinsed with 2mL of Nuclei EZ lysis buffer. This rinse was pooled into the sample for a total of 4mL and then incubated on ice for 5 min. Nuclei were centrifuged at 500 × g for 5 min at 4°C and washed with ice-cold EZ buffer, followed by a second wash in nuclei suspension buffer (NSB) consisting of PBS (Gibco 14,190–144), 1% w/v BSA (Sigma, A9576) and 0.2 U/uL RNase inhibitor (Takara, 2313A).

Isolated cell nuclei from cornea limbal samples were resuspended in 200uL NSB containing 5ug/mL DAPI (ThermoFisher Scientific, D1306). Nuclei integrity was visually inspected under an inverted phase microscope. Isolated nuclei were then filtered through a 35 um nylon mesh cell strainer for nuclei sorting using a FACSAria Fusion sorter (BD Biosciences, 70 um nozzle) at a flow rate between 1–3 to achieve no more than 1000 events per second. Nuclei, defined as DAPI-positive singlets, were sorted into microtubes containing 35 uL NSB. The maximum number of nuclei was sorted from each cornea sample (ranged from 350–5,000 nuclei per sample).

Attempts to count Trypan Blue-positive stained nuclei either prior to sorting or immediately after sorting indicated that the number of nuclei was too low to accurately count without sacrificing a large portion of the cornea samples. Therefore, nuclei were resuspended in the minimal volume required for each step in the isolation procedure without counting or dilution. The average percentage recovery from the FACSAria Fusion sorter was determined from other samples to be 70%, so the final nuclei count for sequencing was calculated by multiplying the number of DAPI^+^ nuclei sorted × 0.7. Sorted nuclei were pelleted by centrifugation (500 × g, 2 min, 4 °C), then carefully resuspended in either an appropriate volume of NSB to yield 100-200k nuclei per mL or a minimum of 20 uL NSB (whichever volume was larger).

Library Construction was performed using the Chromium Next GEM Single Cell 3’ Library & Gel Bead kit v3.1 (10 × Genomics (PN-1000121) with 18 complementary DNA pre-amplification cycles and sequencing on one-high output lane of the NextSeq 500 (Illumina). Sequencing was performed with libraries pooled and loaded in a single lane of an MGISEQ2000-RS sequencer (MGI Tech Co Ltd). The raw data were demultiplexed using an in-house script that allows 1 base mismatch between the barcode sequence and the index sequence data. Data were trimmed to 100b using bbduk (https://sourceforge.net/projects/bbmap/). The fastq snRNAseq data generated in this study has been deposited to Gene Expression Omnibus (GEO) under the entry GSE225355.

FASTQ files were demultiplexed and aligned to the human transcriptome (GRCh38) using the Cell Ranger 6.0.0 pipeline (10X Genomics), with the flag `–include-introns`. We performed all snRNAseq data processing (post-Cell Ranger) in R version 4.2.2. A total of 7,670 cells were called and potential multiplets were eliminated using the Scrublet v.0.2.1 python package^32^. Ambient mRNA was corrected for via DecontX by calling runDectonX() from the singelCellTK package v2. The background value was provided, using the unfiltered Cell Ranger output. Low quality nuclei were identified using the following thresholds (based on pre-DecontX corrected levels): less than 500 unique molecular identifiers (UMIs), less than 500 unique genes or expressing greater than 5% mitochondrial genes. After removing low quality nuclei, 5,667 remained with a median UMI count of 3,157 per nucleus. We used scran v1.26.0 R package to normalize DecontX-corrected UMI counts with pooling-based size factors estimation method, avoiding technical dropout effects^33^. Resulting normalized matrix was imported in Seurat v4.4.0 platform and cell cycle scores for each cell were computed based on expression level of S and G2/M phage marker genes (from the 2019 version)^34^. Principal component analysis (PCA) dimensionality reduction was performed on the top 30% highly variable genes which were identified by decomposing the variance of each gene into its biological and technical components using Scran. Graph-based clustering was carried out on the PCA-reduced expression data using the first 20 principal components and a total of 15 clusters of cells were identified. Corneal cell types were then annotated according to the expression of established markers (KRT14, KRT12, AQP5, DCN and MLANA). Cells were visualized in two dimensions using Uniform Manifold Approximation and Projection (UMAP). We used the Monocle 3 R package for single cell trajectory analysis to investigate LSC differentiation^35^.

### Immunofluorescence and purification of SLC6A6 and ITGβ4 positive cells for clonogenic assay

Human cadaveric eye tissue was acquired from the San Diego Eye Bank from consenting donors that were anonymized, and all confidential patient information was redacted by the eye bank prior to shipment of the tissue. For immunohistochemistry, human eyes were fixed in Davidson’s fixative for 24h before transfer to 70% for dehydration in ascending ethanol concentrations and paraffin embedding. After de-paraffinization, rehydration using descending ethanol concentrations, pressure cooker mediated antigen retrieval, and blocking in 10% goat serum, 5µm paraffin sections were immuno-stained with primary antibodies for SLC6A6 (ab236898—Abcam) and ITGβ4 (ab182120—Abcam) overnight at 4°C. After three consecutive 10min washes in 1X PBS, slides were stained with secondary antibodies for 1h at 37°C, washed again, and mounted with DAPI as a counterstain before fluorescence imaging.

For immuno-labelling, sorting, and in vitro expansion of limbal epithelial progenitors, SLC6A6 and ITGβ4 antibodies were directly conjugated to magnetic beads using a magnetic conjugation kit (ab269890—Abcam) before isolating putative limbal progenitor cells with an easysep magnet (StemCell Technologies). Antibodies were conjugated to magnetic beads according to the manufacturer’s protocol. Cadaveric limbus tissue was incubated in 1X dispase (ThermoFisher) at 37°C to release the corneal epithelium sheet from the limbal biopsy. Sheets were then treated with accutase (ThermoFisher) for 15min at 37°C to generate single cell suspensions for incubation with SLC6A6 and ITGβ4 antibodies conjugated to magnetic beads. Single cell suspensions were then incubated in CnT-Prime media (Cellntec) containing the magnetic bead conjugated antibodies for 30min at room temperature in a 15mL falcon tube. After 10min exposure of the 15mL tube to the easysep magnetic field, the cells bound to either SLC6A6 or ITGβ4 antibodies adhered to the walls of the falcon tube and were washed out and transferred to cell culture plates as SLC6A6 and ITGβ4 enriched populations.

After purification using magnetic beads, purified cells were quantified and then used for clonogenic assay by culturing 500 cells in 6-well culture plates at 37°C with 5% CO_2_ in CnT-Prime media for 10 days. Alternatively, cells were pooled to generate 25,000 purified cells for 8 days to reach confluence for immunolabelling. In brief, 500 purified, or unpurified cells, were cultured in 6-well plates using CnT-Prime media and the presence of colonies were manually quantified using ImageJ software. After 10 days culture, culture plates were fixed with 4% paraformaldehyde and wells were imaged for colony quantification. Colony forming efficiency was calculated by dividing the colony count with the total cells seeded and expressed as a percentage. Holoclone forming assays were then performed by picking individual colonies, dissociating them with TrypLE and plating them in individual wells in 6-well plates for further expansion. Cultures were fixed with 4% paraformaldehyde and imaged for identification of wells with holoclones present. Holoclone formation efficiency was then calculated as the initial colony forming efficiency multiplied by the percentage of wells with a holoclone present after 10 days culture. Data were analyzed using a one-way ANOVA and post-hoc analysis using Tukey’s multiple comparisons testing.

To determine if SLC6A6 and ITGβ4 enriched populations can generate confluent cultures in vitro and maintain expression of basal and differentiated markers, we performed immunocytochemistry of KRT15 (Abcam—ab52816), TP63 (Abcam—ab124762), and KRT12 (Abcam—ab185627) in SLC6A6 and ITGβ4 purified and unpurified cell cultures. Briefly, cells were fixed in 4% paraformaldehyde in PBS pH7.4 for 10min at room temperature before washing three times in cold PBS. Cells were then incubated in 0.1% Triton X-100 to permeabilize cells and washed a further three times in PBS. After blocking in 10% goat serum, cells were incubated in the diluted antibody in 1% BSA in PBST in a humidified chamber overnight at 4°C. After washing three times in PBS, cells were incubated with the secondary antibody in 1% BSA for 1 h at room temperature in the dark, washed a further three times and mounted with hard-set DAPI for imaging and cell quantification in ImageJ. Quantification data were analyzed using a one-way ANOVA and Tukey’s multiple comparisons test post-hoc.

## Data Availability

The snRNA-Seq data generated in this study has been deposited to Gene Expression Omnibus (GEO) under the entry GSE225355.

## References

[1] Cotsarelis G, Cheng SZ, Dong G, Sun TT, Lavker RM (1989). Existence of slow-cycling limbal epithelial basal cells that can be preferentially stimulated to proliferate: implications on epithelial stem cells. Cell.

[2] Di Girolamo N (2015). Tracing the fate of limbal epithelial progenitor cells in the murine cornea. Stem Cells.

[3] Amitai-Lange A (2015). Lineage tracing of stem and progenitor cells of the murine corneal epithelium. Stem Cells.

[4] Parfitt GJ (2015). Immunofluorescence tomography of mouse ocular surface epithelial stem cells and their Niche microenvironment. Investig. Ophthalmol. Vis. Sci..

[5] Schlötzer-Schrehardt U, Kruse FE (2005). Identification and characterization of limbal stem cells. Exp. Eye Res..

[6] Sartaj R (2017). Characterization of slow cycling corneal limbal epithelial cells identifies putative stem cell markers. Sci. Rep..

[7] Haagdorens M (2016). Limbal stem cell deficiency: Current treatment options and emerging therapies. Stem Cells Int..

[8] Pellegrini G (2018). Navigating market authorization: The path holoclar took to become the first stem cell product approved in the European Union. Stem Cells Transl Med.

[9] Joe AW, Yeung SN (2014). Concise review: Identifying limbal stem cells: classical concepts and new challenges. Stem Cells Transl. Med..

[10] Sacchetti M, Rama P, Bruscolini A, Lambiase A (2018). Limbal stem cell transplantation: Clinical results, limits, and perspectives. Stem Cells Int..

[11] Dua HS, Azuara-Blanco A (2000). Limbal stem cells of the corneal epithelium. Surv. Ophthalmol..

[12] Chee KYH, Kicic A, Wiffen SJ (2006). Limbal stem cells: The search for a marker. Clin. Exp. Ophthalmol..

[13] Ebrahimi M, Taghi-Abadi E, Baharvand H (2009). Limbal stem cells in review. J. Ophthalmic Vis. Res..

[14] Ksander BR (2014). ABCB5 is a limbal stem cell gene required for corneal development and repair. Nature.

[15] Mikhailova A (2015). Comparative proteomics reveals human pluripotent stem cell-derived limbal epithelial stem cells are similar to native ocular surface epithelial cells. Sci. Rep..

[16] Mei H, Nakatsu MN, Baclagon ER, Deng SX (2014). Frizzled 7 maintains the undifferentiated state of human limbal stem/progenitor cells. Stem Cells.

[17] Rama P (2010). Limbal stem-cell therapy and long-term corneal regeneration. N. Engl. J. Med..

[18] Barbaro V (2007). C/EBPδ regulates cell cycle and self-renewal of human limbal stem cells. J. Cell. Biol..

[19] Collins PJ (2017). Epithelial chemokine CXCL14 synergizes with CXCL12 *via* allosteric modulation of CXCR4. FASEB J..

[20] Ligocki AJ (2021). Molecular characteristics and spatial distribution of adult human corneal cell subtypes. Sci. Rep..

[21] Li J (2011). S100A expression in normal corneal-limbal epithelial cells and ocular surface squamous cell carcinoma tissue. Mol. Vis..

[22] Ojeda AF, Munjaal RP, Lwigale PY (2013). Expression of CXCL12 and CXCL14 during eye development in chick and mouse. Gene Expr. Patterns.

[23] Hayashi R (2017). Coordinated generation of multiple ocular-like cell lineages and fabrication of functional corneal epithelial cell sheets from human iPS cells. Nat. Protoc..

[24] Sagga N, Kuffová L, Vargesson N, Erskine L, Collinson JM (2018). Limbal epithelial stem cell activity and corneal epithelial cell cycle parameters in adult and aging mice. Stem Cell Res..

[25] Català P (2021). Single cell transcriptomics reveals the heterogeneity of the human cornea to identify novel markers of the limbus and stroma. Sci. Rep..

[26] Collin J (2021). A single cell atlas of human cornea that defines its development, limbal progenitor cells and their interactions with the immune cells. Ocul. Surf..

[27] Chen, J. *et al.* Targeting matrix metalloproteases in diabetic wound healing. *Front. Immunol.* **14**, (2023). 10.3389/fimmu.2023.1089001 PMC9981633 36875064

[28] Li D-Q (2021). Single-cell transcriptomics identifies limbal stem cell population and cell types mapping its differentiation trajectory in limbal basal epithelium of human cornea. Ocul. Surf..

[29] Rong L, Wang L, Shuai Y, Guo H, Liu K (2019). CXCL14 regulates cell proliferation, invasion, migration and epithelial-mesenchymal transition of oral squamous cell carcinoma. Biotechnol. Biotechnol. Equip..

[30] Shellenberger TD (2004). BRAK/CXCL14 is a potent inhibitor of angiogenesis and a chemotactic factor for immature dendritic cells. Cancer Res..

[31] Parapuram SK, Huh K, Liu S, Leask A (2011). Integrin β1 is necessary for the maintenance of corneal structural integrity. Investig. Ophthalmol. Vis. Sci..

[32] Wolock SL, Lopez R, Klein AM (2019). Scrublet: Computational identification of cell doublets in single-cell transcriptomic data. Cell Syst..

[33] Lun, A. T. L., McCarthy, D. J. & Marioni, J. C. A step-by-step workflow for low-level analysis of single-cell RNA-seq data with bioconductor. *F1000Res* **5**, 2122 (2016). 10.12688/f1000research.9501.1 PMC5112579 27909575

[34] Stuart T (2019). Comprehensive integration of single-cell data. Cell.

[35] Cao J (2019). The single-cell transcriptional landscape of mammalian organogenesis. Nature.

